# The development and implementation of an oxygen treatment solution for health facilities in low and middle-income countries

**DOI:** 10.7189/jgh.10.020425

**Published:** 2020-12

**Authors:** Stephen RC Howie, Bernard E Ebruke, Mireia Gil, Beverly Bradley, Ebrima Nyassi, Timothy Edmonds, Sainimere Boladuadua, Senimili Rasili, Eric Rafai, Grant Mackenzie, Yu Ling Cheng, David Peel, Joan Vives-Tomas, Syed MA Zaman

**Affiliations:** 1 Medical Research Council Unit The Gambia at London School of Hygiene & Tropical Medicine, Fajara, The Gambia; 2 Department of Paediatrics: Child & Youth Health, University of Auckland, Auckland, New Zealand; 3 Azimut360 SCCL, Barcelona, Spain; 4 University of Toronto, Toronto, Canada; 5 Cure Kids New Zealand, Auckland, New Zealand; 6 Cure Kids Fiji, Suva, Fiji; 7 Ministry of Health and Medical Services, Suva, Fiji; 8 Medical Research Council Unit The Gambia at London School of Hygiene & Tropical Medicine, Basse, The Gambia; 9 Murdoch Children’s Research Institute, Melbourne, Australia; 10 London School of Hygiene & Tropical Medicine, London, UK; 11 Ashdown Consultants, East Sussex, UK; 12 Liverpool School of Tropical Medicine, Liverpool, UK

## Abstract

**Background:**

Oxygen reduces mortality from severe pneumonia and is a vital part of case management, but achieving reliable access to oxygen is challenging in low and middle-income country (LMIC) settings. We developed and field tested two oxygen supply solutions suitable for the realities of LMIC health facilities.

**Methods:**

A Health Needs Assessment identified a technology gap preventing reliable oxygen supplies in Gambian hospitals. We used simultaneous engineering to develop two solutions: a Mains-Power Storage (Mains-PS) system consisting of an oxygen concentrator and batteries connected to mains power, and a Solar-Power Storage (Solar-PS) system (with batteries charged by photovoltaic panels) and evaluated them in health facilities in The Gambia and Fiji to assess reliability, usability and costs.

**Results:**

The Mains-PS system delivered the specified ≥85% (±3%) oxygen concentration in 100% of 1-2 weekly measurements over 12 months, which was available to 100% of hypoxaemic patients, and 100% of users rated ease-of-use as at least ‘good’ (90% very good or excellent). The Solar-PS system delivered ≥85% ± 3%) oxygen concentration in 100% of 1-2 weekly measurements, was available to 100% of patients needing oxygen, and 100% of users rated ease-of-use at least very good.

Costs for the systems (in US dollars) were: PS$9519, Solar-PS standard version $20 718. The of oxygen for a standardised 30-bed health facility using 1.7 million litres of oxygen per year was: for cylinders 3.2 cents (c)/L in The Gambia and 6.8 c/L in Fiji, for the PS system 1.2 c/L in both countries, and for the Solar-PS system 1.5 c/L in both countries.

**Conclusions:**

The oxygen systems developed and tested delivered high-quality, reliable, cost-efficient oxygen in LMIC contexts, and were easy to operate. Reliable oxygen supplies are achievable in LMIC health facilities like those in The Gambia and Fiji.

Acute respiratory infection (ARI), principally pneumonia, remains a leading cause of death in young children worldwide [1]. Case-management of pneumonia is a key component of the WHO Integrated Management of Childhood Illness (IMCI) strategy and will remain integral to efforts to reduce child mortality [2,3]. WHO guidelines for the management of pneumonia include the appropriate use of oxygen in addition to antibiotic therapy and general supportive care. Oxygen is needed to treat hypoxaemia, a life-threatening feature of severe pneumonia resulting from impaired lung function. Oxygen treatment reduces mortality from severe pneumonia in children substantially, and oxygen is also a core treatment for other respiratory and non-respiratory severe childhood and adult diseases, notably including COVID-19 [4-7].

Medical oxygen is in limited supply in low and middle-income countries (LMIC), a challenge that is exacerbated further by the current COVID-19 pandemic [6,8]. Oxygen is traditionally supplied in high-pressure cylinders, which are expensive, logistically challenging, and often not available [9,10]. Oxygen concentrators provide an alternative to the high cost and logistical problems associated with oxygen cylinders [6,10,11]. Oxygen concentrators are not without limitations, however: maintenance is needed to maximise their life and, importantly, they need a reliable source of power, which is very often not available in LMIC health facilities or to the populations they serve [12,13]. A 2013 review of over 4000 health facilities in 11 countries across sub-Saharan Africa found that only 28% had reliable electricity [14].

A Health Needs Assessment framework was used to address oxygen needs for health facilities in The Gambia [15,16]. A situational analysis showed that oxygen availability was inadequate to meet the needs in most health facilities [17]. An options analysis identified that, while oxygen concentrators had significant advantages over cylinders, currently available solutions were not realistically able to meet the all the challenges of the context, including the need for reliable power [12]. A gap in technology needed to be addressed to reliably give patients and health systems the benefit of oxygen delivered through oxygen concentrators. The goal of this project was to address that gap.

The specific objectives were to develop and field test a concentrator-based oxygen system that would overcome the barriers of unreliable power and maintenance to supply oxygen reliably 24 hours a day, 365 days a year. It was envisaged that continuity of supply would be achieved by combining concentrator technology with power storage and, where needed, an off-grid power source such as solar photovoltaic panels. We aimed to identify the most feasible system that could operate with limited or no mains power and limited maintenance in the target LMIC context, and in doing so we undertook field testing in facilities in both The Gambia and in Fiji.

## METHODS

### Health Needs Assessment framework

A Health Needs Assessment (HNA) framework is the overarching public health methodology we applied to define issues surrounding oxygen treatment, starting in The Gambia, and the options for improving it [15,16,18]. A gap analysis identified the need for a technical solution that would bring the benefits of oxygen concentrators to a context of typically unreliable power supply.

### Technology development approach

Technology development for the project used ‘concurrent or simultaneous engineering’, an approach that considers all aspects of a project in parallel rather than in series, and which facilitates the efficient development and uptake of new technology and in which product attributes are used to evaluate a product throughout the design process [19-21]. We ran experiments simultaneously testing hypotheses that were informing the final design in collaboration with our main suppliers, the end users and the research team.

### Design specification

The design specification called for a system that was modular, designed to supply up to five children and installed in multiples where needed, able to deliver oxygen continuously, with a two-day electricity back-up capacity (Appendix S1 in the **Online Supplementary Document**).

We developed and tested two systems: a mains-connected Power Storage (Mains-PS) system incorporating oxygen concentrator, batteries and electronic control components; and an off-grid Solar-PS system that incorporated an additional photovoltaic array and an online monitoring system. The systems were designed and modeled for performance and refined virtually before production of a prototype. Prototypes were bench-tested to assess actual performance against modeled performance. Successful prototypes were then trialed in the field.

### Initial development and piloting

The early development phase for a PS system was between 2009 and 2014, which involved design, bench-testing and pilot field testing in Basse Health Centre, a 25-bed facility with unreliable electricity serving the Upper River Region of The Gambia [9]. After an assessment of power availability at health facilities in The Gambia to better understand typical power interruption frequencies and durations, a prototype backup power system, supplied by Dulas (UK), was developed and bench-tested at the Medical Research Council Unit, The Gambia (MRCG) [22]. The system consisted of gel sealed batteries, an inverter and a charger, with integrated surge protection. Bench-testing demonstrated that the system could provide oxygen 24 hours per day with as little as 4 hours of charging time, regardless of whether charging time was consecutive hours or dispersed throughout the day. Power interruptions of 2 hours or less had very little effect on the overall charge of the system. For all bench experiments, oxygen concentration was consistently within the required range (≥85% ± 3%) regardless of the power situation (charging from grid or on battery power) [22]. The PS system was then installed at the Basse Health Centre in July 2011 [23]. The PS system was wired to dedicated electrical sockets in the ward, where an Airsep New Life Elite oxygen concentrator (Caire Inc, USA) was connected. The downstream oxygen conduit connected to an Airsep Sureflow 5-flowmeter unit (Caire Inc, USA). Backup from a cylinder supply manifold outside the ward was also connected to the Sureflow and operated by an on-off connector switch. The pilot system functioned to specification, and based on lessons from the pilot period, the design was revised and the components were upgraded to harmonise systems across test sites.

### Field testing

**Figure 1:**
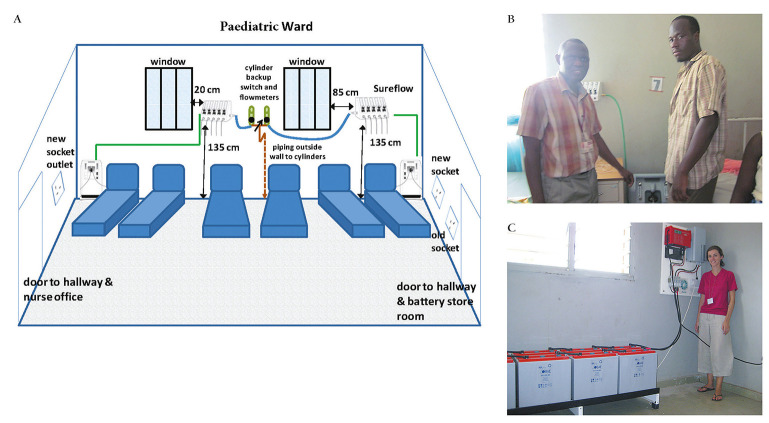
PS system diagram (**Panel A**) and in situ Basse Health Centre (**Panel B** and **Panel C**). **Panel A:** PS system diagram Basse Health Centre. **Panel B:** PS system oxygen concentrator connected to 5-outlet SureFlow (Chart Inc) unit. **Panel C:** PS system electronic control board and battery storage.

We formally field tested the final specification PS systems in paediatric wards at Basse Health Centre, Soma Health Centre and Farafenni Hospital (**Figure 1**) from July 2014 to June 2015. Soma Health Centre has 20 paediatric beds, no oxygen supply, electricity availability for 4-11 hours per day, and serves the Lower River Region of The Gambia; Farafenni Hospital, with 29 paediatric beds, an unreliable oxygen supply, and power available for 20-23 hours per day, serves the North Bank Region of The Gambia. The Solar-PS system was developed in collaboration with Azimut 360 (Barcelona, Spain), and was field tested in Soma Health Centre and Farafenni Hospital (The Gambia), and in Nausori Health Centre, Suva, Fiji, between January 2016 and February 2017. Nausori Health Centre is a busy 6-bedded (child and adult) facility serving the periurban Rewa subdivision of Fiji, and was selected as the initial facility in Fiji for installation because of its high patient burden and its relative accessibility from Suva. Staff training in the detection of hypoxaemia and the use of the Mains-PS and Solar-PS systems was undertaken during implementation and refresher training conducted periodically during the test period.

### Evaluation approach

We measured the oxygen concentration produced by the systems, the availability of oxygen for patients, and the acceptability of the systems to clinical users (nurses and doctors). All admissions had pulse oximetry done routinely during admission.

The success criteria for bench-testing of systems were:

Output ≥85 ± 3% oxygen concentration (the applicable ISO standard [24]) for >90% (acceptable >80%) of monitoring checks, measured at least daily by an oxygen analyser with <1% measurement error range.

The success criteria for the field testing of the systems were as follows:

a) Output ≥85 ± 3%  oxygen concentration for target >90% (acceptable level >80%) of monitoring checks, measured 1-2 weekly by an oxygen sensor with <1% measurement error range.

b) Oxygen available from the systems for up to the number of hypoxaemic children the system was specified to supply for target >90% (acceptable level >80%) of the time, measured by check sheet at admission and daily.

c) Ease of use by actual health staff (rated at least ‘good’ on 5-point scale (poor, fair, good, very good, excellent) by target >80% (acceptable >60%) of users, measured by questionnaire.

The capital and running costs of the systems (comprising all components including concentrators) were measured and compared to the costs of equivalent oxygen delivery with locally available cylinder oxygen. Oxygen costs from the systems were modelled for two differently sized representative health facilities using a cost-modelling tool previously described [12].

Ethical approval for the study was given by the Gambia Government-MRC Joint Ethics Committee (SCC/EC974), and field testing in Fiji approved and coordinated by the Fiji Ministry of Health and Medical Services.

## RESULTS

### Mains-Power Storage (Mains-PS) system

The specification for the Mains-PS system is shown in the Appendix. Bench test data showed that the system worked as specified and could be installed in less than one day. Field testing was carried out between July 2014 and February 2017; data are shown in **Table 1**, and the system in situ is shown in **Figure 1**. A median oxygen concentration of 92.5% (range 82.2%-95.4%) was generated from the system. An SpO_2_ < 90% was recorded on 1156 of 12 863 (8.0%) children (<15 years old) presenting to the study facilities. Oxygen was available from the PS system in all of these instances (100%). Nineteen of 20 (95%) clinical staff users of the system rated its ease of use at least good, and 18/20 (90%) rated it very good or excellent (**Table 2**).

**Table 1:** Power storage (PS) system – oxygen concentration output at maximum flow (5 L/min), and availability of oxygen for clinical care

	Outcome measured	Number/proportion
**Oxygen concentration**	Recordings (No.)	56
	Oxygen concentration output, Median (range)	92.5% (82.2-95.4)
	Proportion of recordings ≥82% ± 3% concentration	100%
**Availability of oxygen to patients:**		
	Pulse oximetry readings (No.)	12863
	Pulse oximetry readings <90% SpO_2_ (No.)	1156
	Pulse oximetry readings <90% for which oxygen was available, No. (%)*	1156 (100%)

SpO_2_ – peripheral capillary oxygen saturation

*Target 90%.

**Table 2:** Power storage (PS) system – ease of use by health staff (5-point scale, “Poor”, “Fair”, “Good”, “Very Good”, “Excellent”)

Outcome measured	Number/Outcome
No. staff	20
No. rating “Poor”	0
No. rating “Fair”	1
No. rating “Good”	1
No. rating “Very Good”	10
No. rating “Excellent”	8
Median rating	Very Good
%≥‘Good’ (Target >80%)	95

### Solar-Power Storage (Solar-PS) system

**Figure 2:**
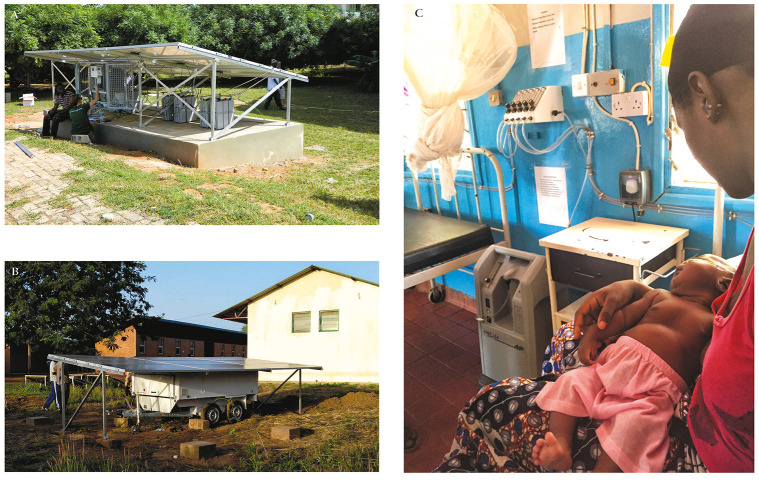
Solar-PS systems in the field. **Panel A:** Standard Solar-PS systems in situ in AFPRC Hospital, Farafenni, The Gambia. **Panel B:** Trailer Solar-PS system installed at Soma Health Centre, Soma, The Gambia.*Wheels were subsequently removed and fence erected to deter theft. **Panel C:** Trailer Solar-PS system supplying oxygen to a child in Soma Health Centre, Soma, The Gambia. *Photograph used with permission of caregiver.

The specification for the Solar-PS system is shown in the Appendix. Bench test data showed that both the standard version (transported in several parts and assembled on site) and trailer version (transported as an integrated unit towed by a vehicle) of the Solar-PS system worked as specified and that both versions could be installed in less than one day. Field testing data are shown in **Table 3** and the system in situ is shown in **Figure 2**.

**Table 3:** Solar-power storage (PS) system – oxygen concentration output at maximum flow (5 L/min), and availability of oxygen for clinical care

Oxygen concentration	Outcome measured	Number/Proportion
	Recordings (No.)	38
	Oxygen concentration output, Median (range)	89.8% (88.0-99.7)
	Proportion of recordings ≥82% ± 3% concentration	100%
**Availability of oxygen to patients:**		
	Pulse oximetry readings (N)	13 862
	Pulse oximetry readings <90% SpO_2_ (No.)	1974
	Pulse oximetry readings <90% for which oxygen was available, No. (%)*	1974 (100%)

SpO_2_ – peripheral capillary oxygen saturation

Field testing showed that the system produced a median oxygen concentration of 89.8% (range 88.0%-99.7%), above the specified minimum of 85±3%. An SpO_2_ < 90% was recorded on 1974 of 13 862 (14.3%) children (<15 years old) admitted to the study facilities. Oxygen was available from the Solar-PS system in all of these instances (100%). All (14/14) clinical staff users of the system rated its ease of use excellent (**Table 4**).

**Table 4:** Solar-power storage (PS) system – ease of use by health staff in participating facilities (5-point scale, “Poor”, “Fair”, “Good”, “Very Good”, “Excellent”), December 2016*

Outcome measured	Number/Outcome
No. staff	15
No. rating ‘Poor’	0
No. rating ‘Fair’	0
No. rating ‘Good’	0
No. rating ‘Very Good’	0
No. rating ‘Excellent’	15
Median rating	Excellent
%≥‘Good’ (Target >80%)	100

*Not yet administered at Nausori Fiji at report date.

**Figure 3:**
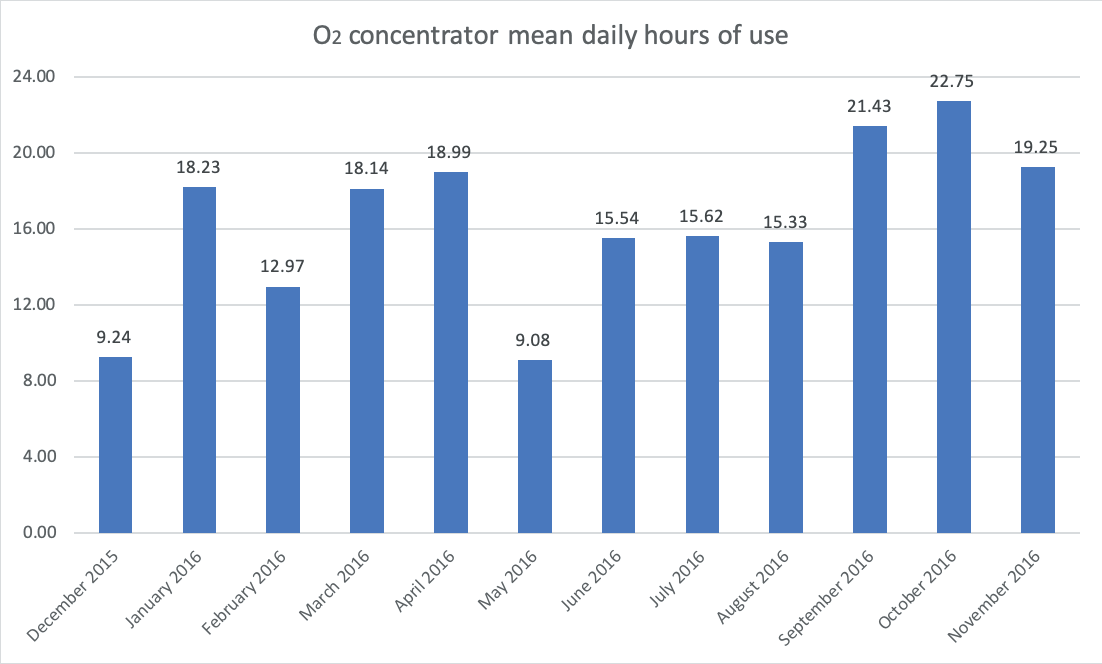
Daily hours of usage of Solar PS oxygen system at Farafenni Hospital during formal 12-month monitoring (December 2015 to November 2016).

Additionally, remote monitoring data were available for the Solar-PS system in Farafenni, which showed over a period of 360 days (from 9 December 2015 to 4 December 2016) that power supply was available 99.5% of the time and batteries were in general in a high state of charge (mean batteries minimum State of Charge:76%). Mean daily hours of use of the O2 concentrator was 16.7 hours per day (**Figure 3**).

### Cost analysis

Equipment costs for the systems were (in US dollars) $9519 for the Mains-PS system and US$20 718 for the standard Solar-PS system, with the trailer-mounted version costing US$26 963 (**Table 5**). The cost of oxygen delivered from the systems, and compared with cylinder-sourced supplies, are shown in **Table 6**. The total costs (capital and running costs) for a standardised paediatric facility of 15 beds and using 500 000 L of oxygen annually (calculated over 5 years) were estimated at US$5515 for the Mains-PS system, US$7322 for the standard Solar-PS system and US$4815 for a cylinder supply with no leakage. We have found that leakage in the Gambian context is common and often significant, and annual cost at 10% leakage is estimated to be US$5350 while at 70% leakage is US$17 832. In Fiji the annual cost for this standardised paediatric facility of the PS system was US$5391, the Solar-PS system US$7322, and the cylinder cost US$10 068 with no leakage (US$11 187 at 10% leakage and US$37 289 at 70% leakage).

**Table 5:** Item capital costs and running costs (US$)* for PS system (including concentrator) supply, solar-PS system (including concentrator) supply, oxygen concentrator powered from mains supply alone, and oxygen cylinder supply

			**PS**	**Solar-PS†**	**Cylinder**	**Concentrator (mains powered)**
**Capital**	Concentrators*		675	675		675
	Patient delivery-associated equipment		495	495	187	495
	Additional equipment		8349	19 548		
	Total equipment cost		9519	20 718	187	1170
**Transport**	Teknon to site		265	265		133
	Shipping		3947	3925		300
**Installation**			484	484		162
**Operating expenses**	Electricity unit cost (per kWh)		22.2 c Gambia, 15.8 c Fiji	0		22.2 c Gambia, 15.8 c Fiji
	Maintenance		2105	1585		842
	Cylinder refill	Gambia			44.52	
		Fiji			51.48	
	Cylinder rent pa	Gambia			0	
		Fiji			1007.28	
	Cylinder transport (per cylinder)				11	

PS – power storage; c – cents

*Exchange rates: 1US$ = Euro0.853 / GBP0.753.

†Standard Solar-PS system. Total equipment costs for the trailer-mounted version were US$26 963.

**Table 6:** Overall oxygen costs (US$) for a standardised paediatric ward* and a standardised all-age health facility† (costs in parentheses are costs without shipping costs included)

			PS	Solar-PS^3^	Concentrator	Cylinder		
						**No leakage**	**10% leakage**	**70% leakage**
Standardised paediatric facility using 0.5 mL/year	Cost per litre oxygen	Gambia	0.0110 (0.0100)	0.0146 (0.0131)	0.0035	0.0096	0.0107	0.0321
		Fiji	0.0108 (0.0092)	0.0146 (0.0131)	0.0033	0.0201	0.0224	0.0755
	Cost per annum	Gambia	5515 (4725.6)	7322 (6533.4)	1762	4815	5350	17832
		Fiji	5391 (4601.6)	7322 (6533.4)	1638	10068	11187	37289
Standardised all-age facility using 1.7 mL/year	Cost per litre Oxygen	Gambia	0.0119 (0.0103)	0.0151 (0.0135)	0.0044	0.0320	0.0356	0.1067
		Fiji	0.0115 (0.0099)	0.0151 (0.0135)	0.0040	0.0679	0.0755	0.2264
	Cost per annum	Gambia	5932 (5142.6)	7566 (6767.4)	2179.0	16006	17785	59282
		Fiji	5755 (4965.6)	7566 (6767.4)	2002	33967	37741	125803

PS – power storage, Solar-PS – solar-power storage

*Standardised paediatric ward used here comprises 15 beds, 500 000L of oxygen used annually, 1286 patients/years (a bed occupancy of approximately 100% across a year – a moderate-high occupancy facility), a 6% prevalence of hypoxemia amongst admissions, and average length of oxygen treatment of 3 days at an average 1.5 L/min flow of oxygen, a total average hospital stay of 5 days, and 5556 oxygen concentrator hours of running annually shared between 2 concentrators (the regulation number for a facility this size, run from one PS or Solar-PS system) with a lifespan of 5 years.

†Standardised all-age facility used here comprises 30 beds, 1.7 mL of oxygen used annually, an average bed occupancy of 67% (reflecting a moderate occupancy facility), half of admissions being children and half adults, a 6% prevalence of hypoxaemia amongst children admitted and a 9% prevalence of hypoxaemia among adults, and average length of oxygen treatment of 3 days at an average 1.5 L/min flow of oxygen for children and 5 L/min for adults, a total average hospital stay of 5 days, and 7906 oxygen concentrator hours of running annually shared between 3 concentrators (the regulation number for a facility this size, run from one PS or Solar-PS system) with a lifespan of 5 years.

For a standard mixed adult-child facility using 1.5 million litres of oxygen annually costs were estimated at US$5932 for the PS system, US$7566 for the Solar-PS system and US$16 006 for a cylinder supply with no leakage (US$17 785 at 10% leakage and $59 282 at 70% leakage). In Fiji the annual cost for this standardised mixed adult-child facility of the PS system was US$5755, the Solar-PS system US$7566, and the cylinder cost US$33 967 with no leakage (US$37 741 at 10% leakage and US$125 803 at 70% leakage).

## DISCUSSION

We found that the two systems developed to address the challenges of achieving a reliable oxygen supply in LMIC health facilities with poor power availability, the Mains-Power-Storage (Mains-PS) system and the Solar-Power storage (Solar-PS) system, delivered good quality oxygen reliably in both Gambian and Fijian settings, and were rated as very user-friendly for staff. These two systems, which use readily available components and are able to be assembled without specialist facilities or expertise, included mains-connected battery power-storage for the Mains-PS system and off-grid battery storage connected to solar power-generating panels for the Solar-PS system. We also found that the costs for oxygen were similar or lower than the existing cylinder supply systems in modelled standardised facilities relevant to the settings of the study. The specifically solar components of the standard Solar-PS system made it around one-third more expensive than the Mains-PS system.

User satisfaction with the Mains-PS and Solar-PS systems was high, and users reported that they generally preferred the new systems to the old cylinder-based supply. The main reasons for this preference were that they did not worry about running out of oxygen, they did not have the logistical complications and transactional costs of procurement, they did not need to move large cylinders, and they were less worried about cylinders falling over and causing a hazard. Training was important to familiarise the staff with the new systems, and refresher training was undertaken periodically to support this. In Fiji users reported that they sometimes found the maximum flow of 5 LPM from the Airsep Elite concentrator used in the systems limiting when treating very sick adults. The usability of oxygen concentrators has also been found to be favourable in other LMIC settings [6,25].

Modelled costs in the smaller-use standardised child facility using PS and Solar-PS systems in the Gambian context were similar to those for a cylinder-based supply, assuming no cylinder leakage, while the costs in the Fijian context were around half those of a cylinder-based supply. The oxygen costs from test systems in a modelled larger-use mixed age facility were around one-half to one-third that of a cylinder-based system in The Gambia, and one-quarter to one-sixth the cost in Fiji. Where mains power is reliable oxygen costs using concentrators alone is around one-third of that from the PS and Solar-PS systems. This becomes highly relevant as wider programmes in a mix of facilities varying in size and power reliability are implemented, and overall cost-efficiency is enhanced.

One barrier to implementation of these and similar systems in LMIC health systems is that they have high initial up-front capital costs. Despite the cost advantages of these systems overall, driven by low running costs, the reorientation of health budgets in LMICs to take advantage of these potential efficiencies can be challenging in practice, and committed leadership and advocacy is required to implement them. Donors can help overcome the hurdle of high capital costs, and often prefer their support to be directed to easily measurable capital items rather than to ongoing running costs. The systems reported here match such donor preferences well.

High use of oxygen tends to increase the comparative cost-efficiency of concentrator-sourced delivery and conversely low use decreases it. The standard facilities used for estimates attempt to reflect different levels of use; however, these standardised facilities involve assumptions that will not apply in all cases. Where a facility uses higher flows of oxygen for shorter periods the estimated costs of oxygen by volume will be lower for the concentrator-based systems than reported here as the cost-efficiency of concentrators increases with increasing output flow. Additionally, our estimates for the standardised facilities assume that one patient at a time is being treated, despite the capacity to treat up to five simultaneously, which will result in higher estimated costs for oxygen from concentrator-based systems. Leakage of oxygen cylinders is routinely observed in our experience in LMIC settings, sometimes at high levels [12]. The comparisons reported assume no leakage from oxygen cylinders, so may underestimate cylinder costs. Underestimation of cylinder costs may also have resulted from no maintenance costs being included for cylinders and related equipment, and, in Fiji, from the comparatively low cylinder transport costs associated with Nausori Health Centre’s high accessibility compared to most locations in Fiji. The cost-efficiency of oxygen delivery methods will depend on the particulars of the facility’s oxygen demand; nevertheless, there is evidence of cost-efficiency from concentrator-based systems in this study.

The health facility staff at the study sites were encouraged to maintain a backup cylinder supply in case there should be problems with the Teknon systems. While these systems proved reliable in the course of field testing, no system is fail-safe and contingencies remain important in all aspects of health systems, which in this case also include trained technicians and adequate spare parts. Cylinders can also meet some needs that the concentrators used in the systems do not, for instance high flow delivery of oxygen (>5LPM), oxygen for transport to a referral facility, and the needs of surgical anaesthesia. Higher flow concentrators, delivering up to 10L/min flow, are available and have been used with success in LMIC settings and can be coupled with the systems reported here [26]. This is relevant particularly for the treatment of adults, who are more likely to require flows above 5 L/min. The Teknon systems can run such concentrators, which have a higher power requirement, and the practical implication of doing so is simply that the system will run from batteries alone for less than the specified two days, which in many settings is not a practical impediment to providing reliable supplies. Concentrators can also be used to support purpose-designed anaesthetic equipment [27].

The logistical capability to supply cylinders reliably, which is challenging, becomes correspondingly more difficult with increasing remoteness. Poor power availability and quality is a reality in many LMIC health facilities, and becomes more likely the more remote a facility is.[14] Universal access to energy by 2030, UN Sustainable Development Goal 7 [3], highlights the importance of electricity, and the WHO has stressed the importance of this for health facilities and the importance of solar solutions to achieve the goal [28,29]. There is more than one approach that can be taken to overcoming these barriers using oxygen concentrators. One is to power the concentrators alone as a core essential medicine, and that is the approach we have taken: while the systems can bear the running of limited other equipment, such as a pulse oximeter and a light, their core aim is to support oxygen supply. Another approach is to power a health facility, or a range of ‘essential’ equipment, and this is an approach being tested in West Africa [30]. Nevertheless, the decision on scope of systems is important and has implications for deliverability, robustness and sustainability. In settings where power is scarce the risks of power being inappropriately diverted to non-core applications is high, and dedicating a power source to one essential piece of equipment using connections not compatible with other equipment reduces this risk. An example of this approach is the solar vaccine fridges used widely in LMICs [31]. Similarly, the Teknon systems are designed for settings in which infrastructure cannot reliably run concentrators connected to mains power and where the running of a range of equipment is a “bridge too far”.

A number of directions for future development are apparent. The Teknon specifications can serve as a platform for further development for more specific clinical and geographical applications, and as suitable alternative components and technology become available. The stipulation that power storage capacity should allow for 2 days running without the need for recharge could be reduced to make the size and cost of the systems smaller, and their portability, and consequent geographical reach, potentially greater. Broader developments in technology that may support the wider roll-out of oxygen systems such as Teknon to reach the least accessible patients include the development of a robust direct current (DC) oxygen concentrator, which would reduce the power requirements, and therefore size and potentially cost, of a system, and advances in battery technology to reduce the size and costs of units needed. A further area for exploration is the inclusion of a low-pressure oxygen storage component, which might have the advantage of reducing the power storage capacity required and increasing the efficiency of use of the concentrator, with the potential disadvantage of a larger footprint and a more complex system, and there has been recent work in this area [32,33]. Reducing mortality and morbidity from hypoxaemic illness is not simply about having a reliable oxygen supply available, but also about patients having access to health services, and then being diagnosed and treated appropriately [34]. Nevertheless, a reliable oxygen supply is the vital starting point.

Oxygen has traditionally been in the “too hard basket” despite it being included in WHO treatment guidelines and Model List of Essential Medicines, but this is no longer the case. It is vital that oxygen be made available to the children, and the adults, that need it. This study shows that the Teknon systems, the specifications of which are made available here, are one means to achieving this aim.

## Additional material

Online Supplementary Document
