# Supplementary material for: The development and implementation of an oxygen treatment solution for health facilities in low and middle-income countries
Source: J Glob Health. 2020 Aug 23;10(2):020425. doi: 10.7189/jgh.10.020425 (PMC7698571; doi:10.7189/jgh.10.020425)
Supplement: Online Supplementary Document [file jogh-10-020425-s001.pdf]

**Appendix: Systems descriptions**

## *Mains-Power Storage (Mains-PS) system*[1]

***System requirements:***

- Modular system
- Specified to operate a 5 LPM concentrator continuously for 24 hours with as few as 4 hours of mains power per day, and to have 2 days electricity backup capacity.
- System components to last 5+ years with little or no maintenance
- Oxygen concentration should be greater than or equal to 85%(+/-3%) at all times.

***System components:***

- 1 x 5 LPM, 350W oxygen concentrator (Airsep Elite, Chart Industries, USA)
- 8 x 6V 350Ah gel sealed valve regulated lead acid (VRLA) deep cycle batteries
- 1 x 1200 VA pure sinewave inverter
- 1 x 48V 50A battery charger
- 1 x battery rack (1.6m x 0.4m footprint)
- 1 x Sureflow 5-way flowmeter assembly (Airsep Elite, Chart Industries, USA)

***System description:***

***The PS system consists of an AC oxygen concentrator powered via a 1200 ampere pure sinewave inverter. The inverter is driven by a bank of eight 6-volt 350 ampere-hour gel sealed, deep-cycle, maintenance-free batteries in series. The batteries are charged by mains power (when available) using a 48 volt, 50 ampere battery charger. The charger, inverter, and outlet are mounted on a control board with connections between all components appropriately fused. Batteries are stored on a battery rack with a footprint of approximately 1.6 meters by 0.4 meters. The system also includes a cylinder backup supply.***

***The standard mode of operation is to have the charger plugged into a mains power outlet with the inverter turned on, and the concentrator on or off as desired. When mains power is available, the batteries will be charging, whether the concentrator is on or not. In the event of a power outage, the system automatically and seamlessly switches to a battery discharge mode.***

***The concentrator is connected to a Sureflow 5-way flowmeter assembly, which has 2 inputs and 5 output flowmeters. These flowmeters can be connected to tubing to supply oxygen to up to 5 children simultaneously at 0.1 to 2 litres per minute flow. As a backup supply, an oxygen cylinder is connected to the second input on the Sureflow. In the event that the concentrator should cease to operate, the cylinder can be manually switched on by turning a knob on the regulator, supplying an alternative flow of oxygen to the Sureflow.***

[1] *bench-test and Basse pilot version*

## *Solar-Power Storage (Solar-PS) system*[2]

***System requirements:***

- Easy-to-use
- Portable
- Specified to operate a 5 LPM concentrator continuously for 24 hours, and to have 2 days electricity backup capacity
- System components to last 5+ years with little or no maintenance
- Oxygen concentration should be greater than or equal to 85%(+/-3%) at all times.
- System autonomy of 16.8 kWh available when fully charged.

***System components:***

- 1 x 5 LPM, 350W oxygen concentrator (Airsep Elite, Chart Industries, USA)
- 9 x 300 Wp solar panels
- PV Panels structure based on Aluminium profiles
- 24 x 2V 350Ah ($C_{10}$-25ºC) gel sealed valve regulated lead acid (VRLA) deep cycle batteries
- 1 x 80A Continuous Maximum Power Point Tracking (MPPT) Solar Charge Controller
- 1 x 1400 VA (48V/230V) pure sinewave inverter/charger
- 8 x 0.4x0.15m battery cages
- 1 x battery monitor
- 1 x online monitoring system
- 1 x GSM router
- 1 x DC protections
- 1 x AC protections
- 1 x alarm system
- 1 x Sureflow 5-way flowmeter assembly (Airsep Elite, Chart Industries, USA)

***System description:***

***The Solar-PS system consists of an AC oxygen concentrator powered via a 1400A pure sinewave inverter/charger. The inverter is driven by a bank of 24 2-volt 350 Ah ($C_{10}$-25ºC) gel sealed, deep-cycle, maintenance-free batteries in series. The batteries are charged by solar power using a 48V, 80A battery charger. The solar charge controller, inverter/charger, battery monitor, online monitoring system and alarm system are mounted on a pre-cabled outdoor ventilated cabinet with connections between all components appropriately fused (including DC and AC surge arresters). Batteries are mounted on 8 portable, ventilated battery cages in groups of 3, with a footprint of approximately 0.4 meters by 0.15 meters each. The system also includes a cylinder backup supply.***

***The standard mode of operation is to have the $O_2$ concentrator plugged to the Solar-PS System, which is capable of powering the O2 concentrator 24 hours a day, even during the worst month of the year (in terms of available solar radiation). The Solar-PS System has 2-days autonomy, although optionally it could be charged by an AC power source (mains or generator) as a support; this capability was not active during the testing.***

***An online monitoring system allows the remote monitoring and adjustment of the system performance.***

***The concentrator is connected to a Sureflow 5-way flowmeter assembly, which has 2 inputs and 5 output flowmeters. These flowmeters can be connected to tubing to supply oxygen to up to 5 children simultaneously at 0.1 to 2 litres per minute. As a backup supply, an oxygen cylinder is connected to the second input on the Sureflow. In the event that the concentrator should cease to operate, the cylinder can be manually switched on by turning a knob on the regulator, supplying an alternative flow of oxygen to the Sureflow.***

---

[2] bench-test and Farafenni modular pilot version
